# Effective Treatment of Established GL261 Murine Gliomas through Picornavirus Vaccination-Enhanced Tumor Antigen-Specific CD8+ T Cell Responses

**DOI:** 10.1371/journal.pone.0125565

**Published:** 2015-05-01

**Authors:** Danielle N. Renner, Fang Jin, Adam J. Litterman, Alexis J. Balgeman, Lisa M. Hanson, Jeffrey D. Gamez, Michael Chae, Brett L. Carlson, Jann N. Sarkaria, Ian F. Parney, John R. Ohlfest, Istvan Pirko, Kevin D. Pavelko, Aaron J. Johnson

**Affiliations:** 1 Neurobiology of Disease Graduate Program, Mayo Clinic, Rochester, MN, United States of America; 2 Department of Immunology, Mayo Clinic, Rochester, MN, United States of America; 3 Department of Neurosurgery, University of Minnesota, Minneapolis MN, United States of America; 4 Summer Undergraduate Research Fellowship, Mayo Clinic, Rochester, MN, United States of America; 5 Department of Neurology, Mayo Clinic, Rochester, MN, United States of America; 6 Department of Neurosurgery, Mayo Clinic, Rochester, MN, United States of America; 7 Department of Radiation Oncology, Mayo Clinic, Rochester, MN, United States of America; University of Florida, UNITED STATES

## Abstract

Glioblastoma (GBM) is among the most invasive and lethal of cancers, frequently infiltrating surrounding healthy tissue and giving rise to rapid recurrence. It is therefore critical to establish experimental model systems and develop therapeutic approaches that enhance anti-tumor immunity. In the current study, we have employed a newly developed murine glioma model to assess the efficacy of a novel picornavirus vaccination approach for the treatment of established tumors. The GL261-Quad system is a variation of the GL261 syngeneic glioma that has been engineered to expresses model T cell epitopes including OVA_257–264_. MRI revealed that both GL261 and GL261-Quad tumors display characteristic features of human gliomas such as heterogeneous gadolinium leakage and larger T2 weighted volumes. Analysis of brain-infiltrating immune cells demonstrated that GL261-Quad gliomas generate detectable CD8+ T cell responses toward the tumor-specific K^b^:OVA_257–264_ antigen. Enhancing this response via a single intracranial or peripheral vaccination with picornavirus expressing the OVA_257–264_ antigen increased anti-tumor CD8+ T cells infiltrating the brain, attenuated progression of established tumors, and extended survival of treated mice. Importantly, the efficacy of the picornavirus vaccination is dependent on functional cytotoxic activity of CD8+ T cells, as the beneficial response was completely abrogated in mice lacking perforin expression. Therefore, we have developed a novel system for evaluating mechanisms of anti-tumor immunity *in vivo*, incorporating the GL261-Quad model, 3D volumetric MRI, and picornavirus vaccination to enhance tumor-specific cytotoxic CD8+ T cell responses and track their effectiveness at eradicating established gliomas *in vivo*.

## Introduction

Glioblastoma (GBM) is among the most lethal of cancers, with a median survival of less than 15 months post-diagnosis despite aggressive, multi-modal treatment[1]. Recurrent tumor infiltration of previously healthy brain tissue occurs in greater than 95 percent of patients within five years of initial treatment, underlining the need for effective immunotherapeutic approaches[1, 2]. Central to the aim of establishing protective immunity is the development of novel strategies that both enhance CD8+ T cell recognition of tumor-associated antigens and promote infiltration of these cells into the central nervous system (CNS)[3–5].

The relevance of tumor-specific CD8+ T cell enhancement in human gliomas has been demonstrated in a number of clinical trials. Biopsy samples isolated from GBM patients have confirmed the presence of CD8+ T cells forming immunological synapses with tumor cells[6]. Furthermore, vaccination with autologous tumor lysate-pulsed dendritic cells (DC) and toll-like receptor (TLR) agonists in a phase I clinical trial resulted in the enhancement of tumor-infiltrating CD8+ T cells and increased survival among a subset of patients[3]. Similarly, a phase II clinical trial using autologous tumor lysate-pulsed DCs demonstrated a logarithmic correlation between clinical outcome and the magnitude of T cell responses, as measured by IFN-γ production[7]. The results of these studies demonstrate the potential of immunotherapeutic approaches aimed at promoting CD8+ T cell responses in improving outcomes for glioma patients.

Consistent with clinical trials, studies in murine tumor models have also demonstrated a correlation between tumor antigen-specific CD8+ T cell activation, reductions in tumor burden, and prolongation of overall survival[5, 8–11]. Notably, combined treatment of GL261 gliomas with a TGF-β neutralizing antibody to reduce immunosuppression and vaccination with glioma-associated antigens resulted in increased tumor-specific, IFN-γ producing CD8+ T cells and improved survival[11]. While the above findings demonstrate that immunotherapeutic targeting of CNS tumors is safe and has the potential for generating detectable anti-tumor immune responses, they also underline the necessity for more effective immunotherapeutics and disease models in which to investigate them. Paramount in this effort is the ability to generate CD8+ T cell responses that can overcome the immunosuppressive environment associated with gliomas.

Introducing GL261 syngeneic gliomas into C57BL/6 mice is a leading pre-clinical model system for evaluating novel immunotherapeutic approaches[12–15]. Detailed characterization of this model has demonstrated a number of histopathologic features relevant to human GBM, including perivascular proliferation, psuedopalisading necrosis, and glioma cell invasion beyond the bulk tumor [12, 16, 17]. Additionally, this model provides the distinct advantage of an immune-competent host, with inflammation evident in the brains of GL261-bearing mice. However, observed CD8+ T cell responses are ineffective at eradicating the glioma cells and mice ultimately succumb to the tumor. Glioma cell lines incorporating model epitopes have been developed which enable the tracking of robust tumor antigen-specific CD8+ T cells and optimization of immunotherapies designed to enhance these responses[8, 18]. A recent study using the engineered GL26 glioma cell line expressing ovalbumin demonstrated successful confocal microscopic imaging of tumor antigen-specific CD8+ T cells localized within the tumor area following immunotherapeutic treatment, which corresponded to reductions in tumor burden and mortality[8]. Thus, while their contributions to anti-tumor immunity are readily demonstrated, defining the mechanisms by which CD8+ T cells are beneficial in tumor eradication remains a critical avenue of research.

Picornavirus vector delivery of tumor-specific antigens represents a promising approach for generating immune responses that can overcome the immunosuppressive environment associated with gliomas. Intracranial infection of C57BL/6 mice with Theiler’s murine encephalomyelitis virus (TMEV) results in a robust virus epitope-restricted CD8+ T cell response that clears the virus after approximately 28 days[19, 20]. Furthermore, the small TMEV genome can be easily engineered to encode tumor antigens, and the resulting recombinant viruses are effective at eliciting tumor antigen-restricted CD8+ T cell responses. Capitalizing on this capacity, this novel vaccination strategy was recently employed in both B16-OVA melanoma and Her2/neu+ breast cancer models[21, 22]. Vaccination of a B16-Ova melanoma with recombinant, OVA-expressing TMEV resulted in a robust antigen-restricted CD8+ T cell response corresponding to a delay in tumor growth and improved survival[21]. Subsequent work targeting the weakly immunogenic endogenous breast tumor antigen Her2/neu also proved effective at generating antigen-restricted CD8+ T cell responses and delaying tumor progression[22]. For the above reasons, the high levels of CD8+ T cells infiltrating the brain, and the lack of long-term disability following viral infection, TMEV and related picornaviruses are strong candidates for eliciting immunity against CNS tumors[19, 23].

The present study utilizes the GL261 and engineered GL261-Quad gliomas to employ novel 3D volumetric MRI in conjunction with analysis of endogenous and picornavirus vaccination-induced CNS-infiltrating immune cells. As such, this work serves as a template for analysis of tumor volume *in vivo* during the course of immunotherapeutic treatment and establishes the utility of picornavirus-based vaccination for the treatment of established gliomas.

## Materials and Methods

### GL261 Cell Culture and Tumor Implantation

The GL261-Quad cell line was generated and maintained as described by Ohlfest *et al*.[18]. This stable cell line expresses the model antigens human gp100_25–33_, chicken OVA_257–264_, chicken OVA_323–339_, and the mouse alloantigen I-Ea_52–68_, with translation of these epitopes detected by DsRed fluorescence [18, 24]. Tumors were implanted into six to twelve week old female C57BL/6 mice (The Jackson Laboratory #000664) or perforin deficient mice (The Jackson Laboratory #002407). Anesthetized mice were stereotactically injected in the right striatum with 6.0 x 10^4^ to 1.0 x 10^5^ GL261 or GL261-Quad syngeneic glioma cells in a total volume of 1μL of physiologic saline at a rate of 0.2μL/min. Injection coordinates were 2.5mm lateral, 0.5mm anterior of bregma, at a depth of 3mm from the cortical surface. Negative controls include sham surgery with injection of PBS and un-manipulated animals. All experimental procedures involving animals were carried out in strict accordance to guidelines and protocols approved by the Mayo Clinic Institutional Animal Care and Use Committee and all efforts were made to minimize suffering throughout the duration of each experiment.

### MRI Acquisition and Analysis

MRI was performed using a Bruker DRX-300 (300 MHz 1H) 7 Tesla vertical-bore small animal imaging system (Bruker Biospin, Billerica, MA) similarly to published protocols[25, 26]. Throughout imaging, mice were anesthetized by inhalation of 3–4% isofluorane in air and their respiratory rate monitored. For T1 weighted imaging, mice were administered gadolinium intraperitoneally (i.p.) at a dose of 100mg/kg and imaged after a 15 minute delay. T2 weighted MRI: RARE pulse sequence, repetition time (TR) = 1500ms, echo time (TE) = 70ms, RARE factor: 16, field of view (FOV): 3.2x1.92x1.92cm, matrix: 256x128x128. T1 weighted MRI: MSME, TR: 300ms, TE: 9.5ms, FOV: 4.0x2.0x2.0cm, matrix: 192x96x96. 3D tumor volumes were determined using Analyze 11.0 software (Biomedical Imaging Resource, Mayo Clinic, Rochester, MN) by a blinded observer. Tumor area was delineated on each axial slice and 3D tumor volume was generated from the acquired measurements.

### Bioluminescence Imaging and Analysis

*In vivo* bioluminescence imaging (BLI) was performed using an IVIS200 system (Xenogen Corp., Alameda, CA) coupled to a PC running Living Image 2.6 software. Mice were administered D-luciferin (Gold Biotehnology, St. Louis, MO) at a dose of 150mg/kg in a volume of 200uL (i.p.). Throughout imaging, anesthesia was maintained using a nose cone delivery system administering 1–2% isofluorane. Images were acquired with an exposure time of 10 seconds with F-stop = 1. Greyscale photographic surface images were collected and overlayed with pseudo-color images representing distribution of emitted photons. Signal intensity was quantified as photons/second in a designated region of interest prescribed over the mouse head.

### Hematoxylin and Eosin Staining

Fresh frozen brains were embedded in OCT compound (Tissue-Tek). Sections 6μm thick were fixed in 10% neutral buffered formalin for 10 minutes. Next, sections were stained with filtered Gill 3X hematoxylin (Thermo, Rockford, IL) for 1 minute, differentiated with acid alcohol, and blued with ammonia water for 15 seconds. Between each step, sections were thoroughly washed with water. Slides were counterstained with Eosin-Phloxine (Sigma, St. Louis, MO) for 30 seconds and dehydrated with 95% alcohol followed by absolute alcohol. Slides were cleared by rinsing in xylene 2 x 5 minutes and covered with Permount mounting media (Thermo).

### Flow Cytometry

Flow cytometry samples were run on a BD LSR II flow cytometer and analyzed with FACSDiva Software (BD Biosciences, San Jose, CA). For analysis of CNS infiltrating immune cells, whole brains were homogenized, collagenase digested, and centrifuged against a Percoll gradient to isolate immune cells as described in detail[19, 25]. The composition of isolated immune cells was determined by staining with antibodies recognizing CD45 (BD 557235), CD8 (BD 552877), CD4 (BD 553730) CD11b (BD 557396) and GR-1 (BD 557661). Tumor antigen-specific CD8+ T cells were determined by staining with K^b^:OVA_257–264_ MHC-peptide tetramers constructed within our laboratory.

### Construction of MHC-peptide tetramers

MHC-peptide tetramers were constructed following published protocols[19, 27]. Briefly, H-2K^b^ class I molecule was folded with the SIINFEKL peptide in the presence of β2m. Monomers were biotinylated using a BirA biotin ligase kit (Avidity, Aurora, CO) and purified over a Mono-Q cation-exchange column. Tetramers were produced by mixing monomers with allophycocyanin-conjugated streptavidin (Sigma) to a molar ratio of 4.0:0.9 and purified by size-exclusion on an S-200 column.

### Treatment of GL261-tumor bearing mice

Recombinant TMEV Xho1-OVA8 virus was generated as described by Pavelko *et al*.[22]. Two weeks post-tumor implantation, tumor load was assessed by BLI and mice with established tumors were divided into treatment groups based on overall equivalent tumor load. Mice were inoculated intracranially (i.c.) in the non-tumor bearing hemisphere with 2 x 10^4^ PFU of control TMEV-wt or recombinant TMEV Xho1-OVA8 picornavirus. Alternatively, mice were inoculated i.p. with 2 x 10^5^ PFU of picornavirus. Mice without detectable tumor two weeks post-implantation were excluded from study.

### Survival

Mice were monitored and euthanized at the time when they began to display symptoms of morbidity, including hunched posture, reduced mobility, and weight loss, in accordance with Mayo Clinic IACUC standards.

### RT-PCR

Total RNA was extracted from homogenized whole brains bearing GL261-Quad gliomas using TRIzol Reagent and reverse transcribed using the Superscript cDNA synthesis kit (Invitrogen, Carlsbad, CA). cDNA was amplified using the Fast SyBR Green Master Mix Kit (Applied Biosystems) using primers generated for mouse actin (5’-CTGGCACCACACCTTCTACAATGAGCTG-3’ and 5’-GCACAGCTTCTCTTTGATGTCACGCACGATTTC-3’) and the Quad cassette (5’-CAGGATTGGCTGGGCGTCAGTCGACA-3’ and 5’- TGGGCCTCGAAGGAGGCGAACTTG-3’). Mean crossing point for each transcript was determined and normalized to actin (deltaCt). DeltaCt values were then used to calculate fold-change relative to actin using the 2^-[delta][delta]Ct method [28]. Data are reported as expression relative to untreated controls.

### Statistical Analysis

Bioluminescence intensity (p/sec), tumor volume (mm^3^), and percent of immune cell subsets are presented as mean ± SEM. All experiments were performed in duplicate or triplicate, with representative data presented. For comparisons of two groups, significant differences were determined using Student’s t-test or using Mann-Whitney Rank Sum Test if the data was found to not follow a normal distribution. For comparisons between multiple groups, a One-Way Analysis of Variance (ANOVA) was followed by a Student-Newman-Keuls (SNK) post-hoc analysis. Differences in survival were determined using the log-rank (Mantel-Cox) test. All calculations were performed using SigmaStat software (SYSTAT Software Inc). Significance is denoted as *p<0.05, **p<0.01, ***p<0.001.

## Results

### Validation of the GL261-Quad model system via in vivo imaging and histopathology

**Fig 1:**
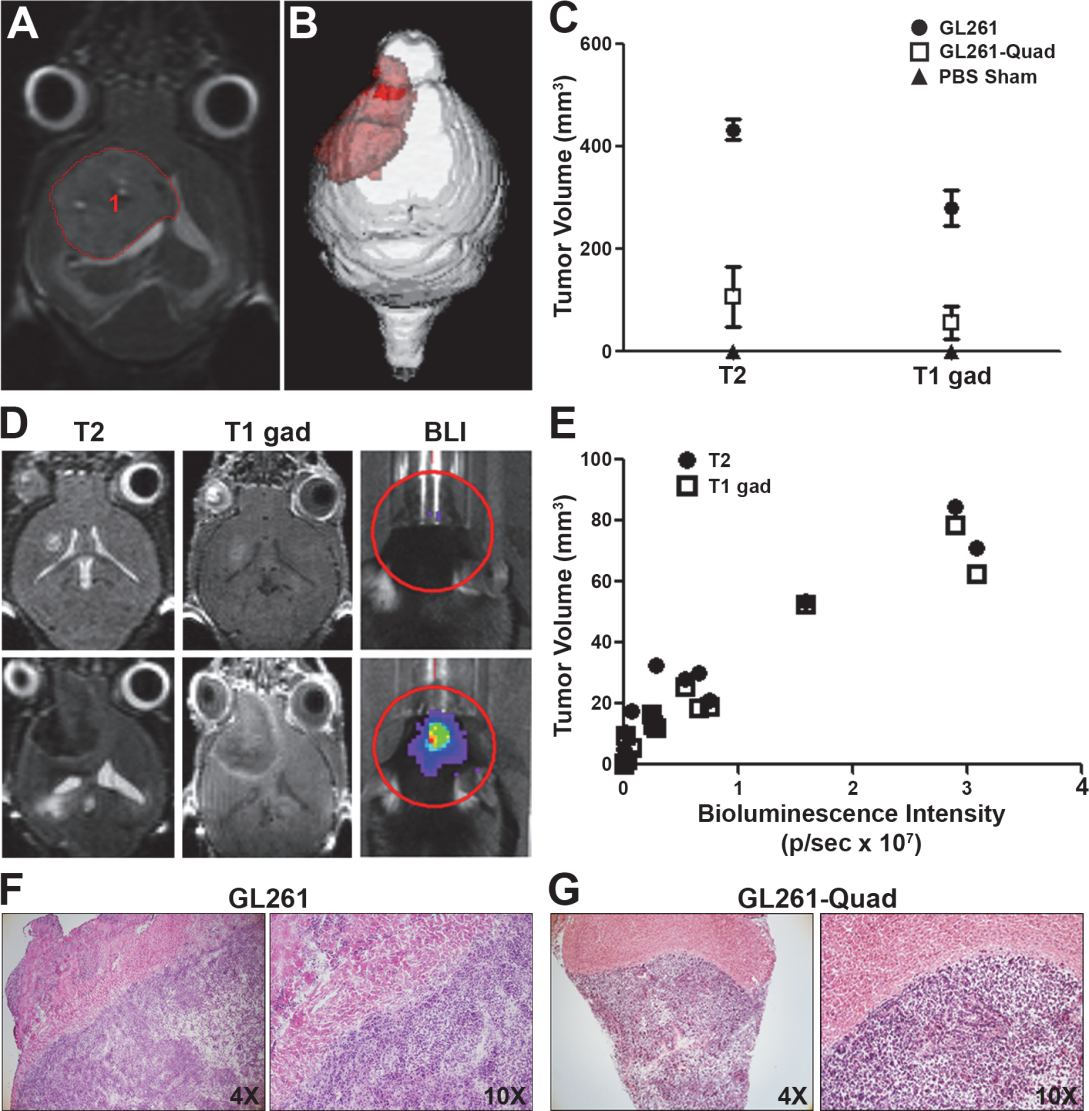
Volumetric analysis of GL261 gliomas through quantification of T2 and T1 gadolinium enhanced MRI *in vivo*. (A,B) Volumetric analysis and 3D rendering of a representative live mouse bearing a GL261 glioma as visible by T2 weighted MRI using Analyze 11.0 software. (C) Quantification of mean tumor volumes demonstrates larger values from T2 weighted MRI than T1 gadolinium-enhanced MRI for both GL261 (N = 3 mice) and GL261-Quad (N = 4 mice) gliomas 4 weeks post tumor injection compared to PBS sham controls (N = 2 mice). (D) T2 weighted and T1 gadolinium-enhanced MRIs with corresponding bioluminescence images of representative small and large GL261-Quad gliomas. (E) Tumor volumes obtained from MRI plotted against bioluminescence intensity demonstrate a strong correlation for both T2 weighted (R^2^ = 0.9045) and T1 gadolinium-enhanced (R^2^ = 0.9296) imaging (N = 15 mice/group). (F,G) Representative H&E stained sections at 4X and 10X magnification of cortical brain tissue from mice bearing GL261 or GL261-Quad gliomas demonstrate similarities in histopathologic features of the two models.

The vast majority of human GBM presents with a greater volume when assessed with T2 weighted MRI than with T1 gadolinium-enhanced MRI[29, 30]. To determine the relative presentation of GL261 and GL261-Quad gliomas, both T2 and T1 gadolinium-enhanced MRI were performed in the same set of mice harboring 4-week established gliomas, a timepoint determined to be just prior to the onset of symptoms for GL261 tumors. Analysis of tumor size was achieved using Analyze 11.0 software (Fig 1A), which allows for volumetric measurements of 3D renderings of the gliomas (Fig 1B). Tumor volumes obtained from T2 weighted MRI were consistently larger than those from T1 gadolinium-enhanced MRI in both the GL261 (N = 3 mice) and GL261-Quad (N = 4 mice) tumor sets (Fig 1C). At this timepoint, mice bearing GL261 gliomas presented with larger tumors than animals bearing GL261-Quad gliomas, indicative of a potential endogenous inflammatory response directed toward the incorporated tumor antigens (Fig 1C). Nevertheless, despite the expression of model T cell epitopes, the majority of mice implanted with the GL261-Quad cells failed to clear them and ultimately succumbed to the tumor. Importantly, GL261-Quad tumor sizes as determined by BLI showed a strong correlation with both T1 gadolinium-enhanced (R^2^ = 0.9296) and T2 weighted (R^2^ = 0.9045) MRI, validating the MR imaging parameters and confirming maintained luciferase expression in the engineered GL261-Quad model system (Fig 1D and 1E, N = 15 mice/group). Furthermore, H&E staining of cortical brain sections of representative glioma-bearing mice validated similarities in the histopathology of GL261 and GL261-Quad gliomas *in vivo* (Fig 1F and 1G)

### GL261-Quad gliomas elicit MHC class I restricted CD8+ T cell responses against tumor-specific antigen

**Fig 2:**
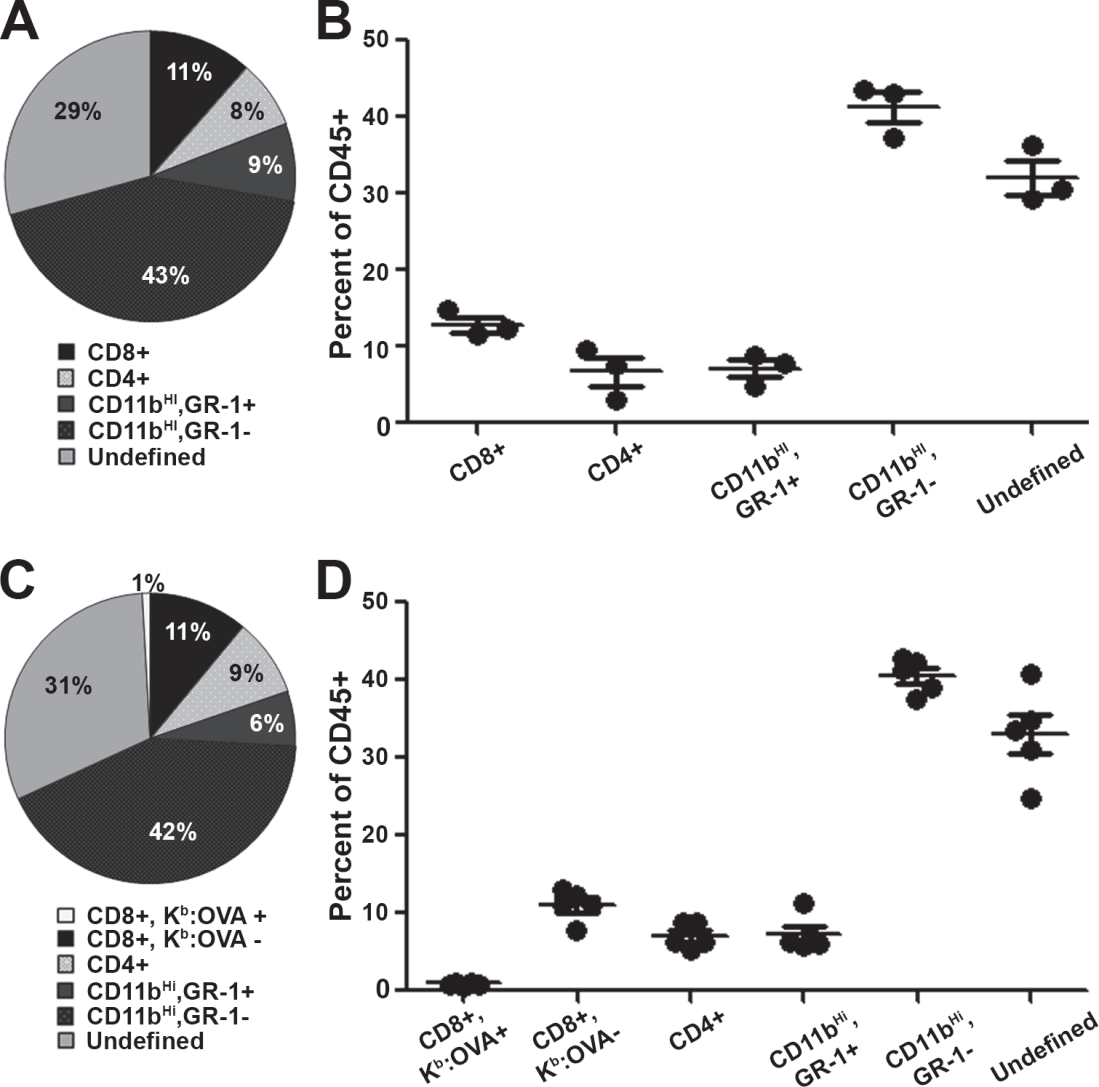
GL261-Quad gliomas elicit an endogenous K^b^:OVA_257–264_ restricted CD8+ T cell response. Immune cells from whole brain tissue of glioma-bearing mice were isolated and gated based on CD45 expression. CD45^Hi^ cells were further analyzed for CD8, CD4, CD11b, GR-1 and K^b^:OVA_**257–264**_ tetramer positivity. (A) The inflammatory profile of a representative GL261-bearing mouse. (B) Relative percentages of immune cell subsets in GL261 glioma-bearing mice confirm defined populations of CD8+, CD4+, and CD11b+,GR-1+ cells (N = 3 mice). (C) The inflammatory profile of a representative GL261-Quad glioma-bearing mouse. (D) K^b^:OVA_**257–264**_ tetramer staining shows the presence of endogenous tumor antigen-restricted CD8+ T cell responses in the absence of treatment in mice bearing GL261-Quad gliomas (N = 5 mice).

To determine the extent to which the immune response against GL261-Quad gliomas resembles that seen in mice bearing GL261 gliomas, CNS infiltrating immune cells were isolated and analyzed for their inflammatory profile. Following inoculation with GL261 (Fig 2A and 2B, N = 3 mice) or GL261-Quad cells (Fig 2C and 2D, N = 5 mice), mice were monitored for four or five weeks, respectively, at which point they displayed symptoms of tumor burden. Among the leukocyte population isolated from whole brain tissue, CD45^Hi^ cells were further analyzed for the expression of CD8 (killer T cells), CD4 (helper T cells, T regulatory cells), CD11b (α_M_-integrin, granulocyte/macrophage marker), and GR-1 (myeloid differentiation antigen). The CD11b^Hi^ subset was consistently the dominant cell type as a percentage of CD45 expressing cells in all mice observed. A distinct subset of these cells stained positively for both the CD11b and GR-1 markers, indicative of a potential myeloid-derived suppressor cell (MDSC) phenotype[31]. All mice displayed defined populations of CD8+ and CD4+ T cells, but at a lower frequency than CD11b^Hi^ cells. Importantly, populations of CD8+, CD4+, CD11b^Hi^ and CD11b^Hi^,GR-1+ cell subsets as a percentage of CD45^Hi^ cells infiltrating the CNS presented similarly between the GL261 and GL261-Quad samples, demonstrating that the presence of model antigens alone does not significantly alter the endogenous inflammatory response (Fig 2). PBS sham surgery controls presented with minimal inflammatory cell infiltration, with only 293 ± 71 CD45^Hi^ cells per 100,000 events collected compared to 13,765 ± 10,576 CD45^Hi^ cells per 100,000 events collected from tumor-bearing mice. Additionally, PBS sham surgery mice displayed minimal percentages of both CD8+ and CD4+ T cells (data not shown).

To evaluate the tumor-specific CD8+ T cell response, CNS-infiltrating immune cells were additionally stained with K^b^:OVA_257–264_ tetramers. All mice bearing GL261-Quad gliomas displayed measurable K^b^:OVA_257–264_ epitope restricted CD8+ T cells within the brain (Fig 2C and 2D), as compared to both PBS sham surgery and parental GL261 glioma controls. Thus, tumor antigen-specific CD8+ T cell responses are elicited spontaneously, without vaccination, against implanted GL261-Quad gliomas. However, this response is insufficient to prevent progression of the tumor.

### Vaccination with an engineered picornavirus significantly increases levels of tumor antigen-specific CD8+ T cells infiltrating the CNS

**Fig 3:**
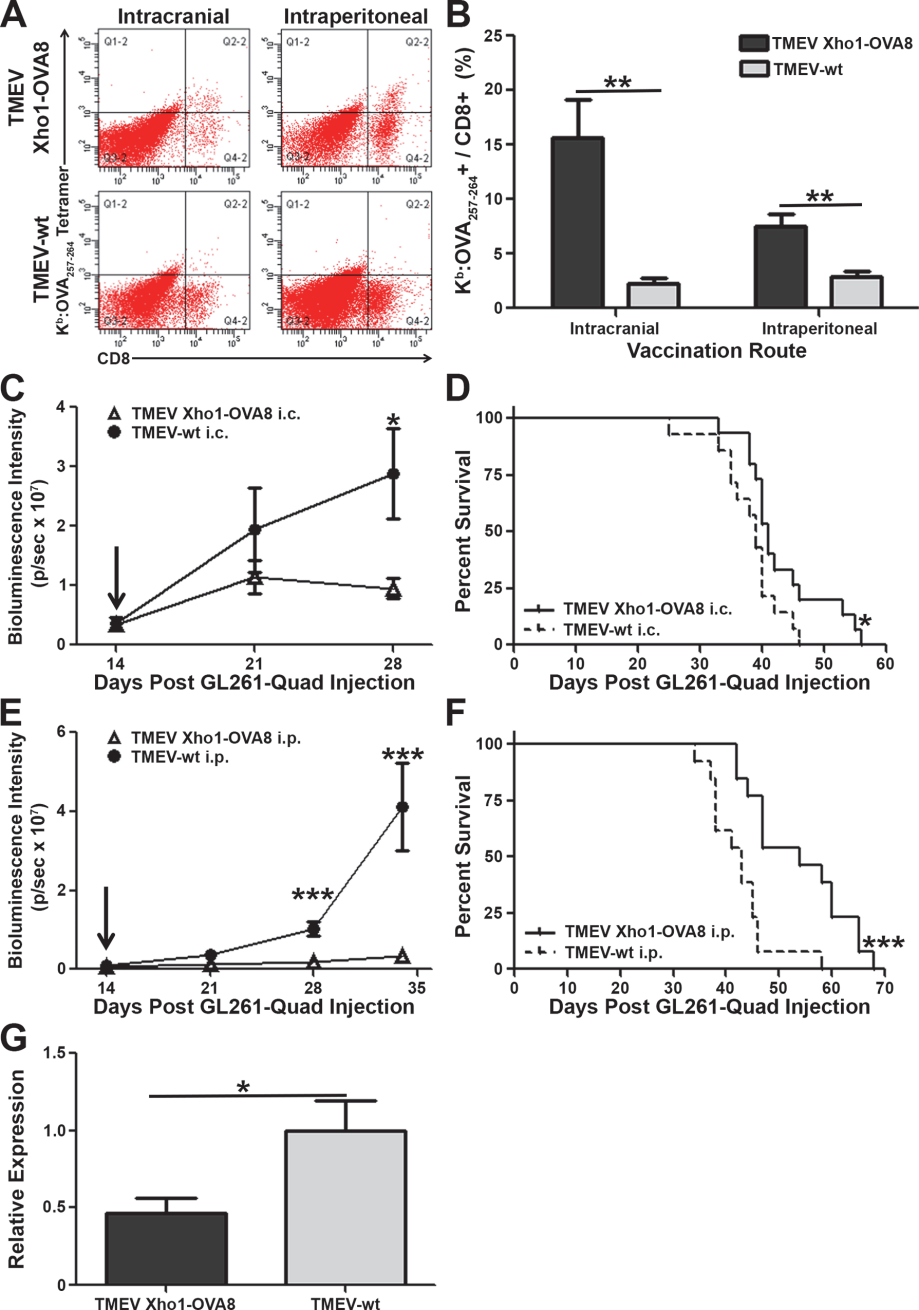
Vaccination with TMEV Xho1-OVA8 enhances tumor antigen-restricted CD8+ T cell responses in the CNS, delays tumor progression, and extends survival in mice bearing GL261-Quad gliomas. Two weeks post-GL261-Quad tumor introduction, mice were treated with control or recombinant picornavirus. (A,B) At five weeks, brain infiltrating lymphocytes were isolated, gated based on CD45 expression, and analyzed for CD8 and K^b^:OVA_**257–264**_ tetramer positivity. (A) Representative FACS plots and (B) mean percent of K^b^:OVA_**257–264**_+ cells per CD8+ cells demonstrate a significant increase in tumor antigen-restricted CD8+ T cells infiltrating the brain following intracranial (N = 9) or intraperitoneal (N = 10 mice) TMEV Xho1-OVA8 vaccine administration compared to TMEV-wt. Percent of K^b^:OVA_**257–264**_+ / CD8+ calculated as Q2-2/(Q2-2+Q4-2)*100. (C,E) Mean bioluminescence intensity (p/sec) of GL261-Quad glioma-bearing mice treated with TMEV-wt or recombinant TMEV Xho1-OVA8 picornavirus. Mice receiving (C) intracranial (N = 15 mice) or (E) intraperitoneal (N = 13 mice) TMEV Xho1-OVA8 treatment displayed significantly delayed progression of established gliomas compared to TMEV-wt treated controls. (D,F) Delayed tumor progression is accompanied by a significant increase in survival for mice treated with TMEV Xho1-OVA8 via (D) intracranial or (F) intraperitoneal administration. Arrow denotes time of vaccine administration. (G) RT-PCR analysis of RNA isolated from brains of glioma-bearing mice demonstrates a significant reduction in transgene expression following treatment with TMEV Xho1-OVA8 compared to TMEV-wt treated controls. Error bars indicate SEM. * denotes p<0.05, ** denotes p<0.01, ***denotes p<0.001.

While an endogenous K^b^:OVA_257–264_ specific CD8+ T cell response was detected toward the GL261-Quad gliomas, this response lacked sufficient effectiveness in clearing established tumors (Fig 2). To determine the extent that picornavirus vaccination could enhance tumor antigen-restricted CD8+ T cell responses in the brain, mice were implanted with GL261-Quad tumors and allowed to progress two weeks. Tumor sizes were then determined by bioluminescence imaging (BLI) and mice were divided into two groups containing overall equivalent tumor loads. In one group, mice harboring equivalent tumor loads were then vaccinated by a single intracranial (i.c.) injection into the non-tumor-bearing hemisphere of the brain with TMEV expressing OVA_257–264_, TMEV Xho1-OVA8 (N = 9 mice), or TMEV-wt (N = 6 mice) (Fig 3A and 3B). The non-tumor-bearing hemisphere was selected due to the highly immunosuppressive environment associated with the gliomas, which could potentially overwhelm any beneficial CD8+ T cell response generated[32]. In a parallel experiment, mice were vaccinated by a single intraperitoneal (i.p.) injection of TMEV Xho1-OVA8 (N = 10 mice) or TMEV-wt (N = 9 mice) (Fig 3A and 3B). Five weeks post-tumor implantation (3 weeks post vaccination), mice were euthanized, brain infiltrating lymphocytes isolated, and CD8+ T cells evaluated for K^b^:OVA_257–264_ tetramer positivity. As compared to TMEV-wt administration, a single intracranial vaccination with TMEV Xho1-OVA8 resulted in significantly increased K^b^:OVA_257–264_ antigen-specific CD8+ T cell infiltration, with approximately 15% of the CD8+ T cell response specific for the OVA_257–264_ antigen (Fig 3B, p = 0.003). Similarly, a single i.p. vaccination with TMEV Xho1-OVA8 resulted in nearly 10% of CNS-infiltrating CD8+ T cells displaying K^b^:OVA_257–264_ antigen-specificity (Fig 3B, p = 0.003). In contrast, mice vaccinated with TMEV-wt by intracranial or peripheral injection presented with levels of K^b^:OVA_257–264_ tetramer positivity comparable to endogenous responses seen in untreated animals bearing GL261-Quad tumors. No differences were detected in the frequency of CD4+ T cells, NK cells, B cells, or macrophages/monocytes infiltrating the brain between the two treatment groups (data not shown).

### Enhanced CD8+ T cell responses toward GL261-Quad gliomas are accompanied by delayed tumor outgrowth and prolonged survival

To determine if a reduction in tumor burden and prolonged survival accompanied enhancement of anti-tumor CD8+ T cell responses, tumor size and animal morbidity were monitored in both TMEV Xho1-OVA8 and TMEV-wt treated mice. GL261-Quad gliomas were again allowed to progress two-weeks before being ranked and assigned to groups of equivalent tumor burden. Intracranial vaccination with TMEV Xho1-OVA8 (N = 15 mice) resulted in markedly delayed tumor progression, reaching statistical significance within 14 days of vaccine administration (Fig 3C, p = 0.03). TMEV-wt (N = 14 mice) was ineffective at controlling tumor growth, with mice becoming symptomatic and requiring euthanasia before the fifth week, precluding further imaging. The observed reduction in tumor burden was accompanied by significantly prolonged survival (Fig 3D, p = 0.041). Intraperitoneal vaccination similarly slowed tumor progression in TMEV Xho1-OVA8 treated mice compared to controls (Fig 3E, N = 13 mice/group, p<0.001). Median survival of mice receiving TMEV Xho1-OVA8 i.p. was 54 days, compared to 43 days observed for TMEV-wt treated controls (Fig 3F, p<0.001). Semi-quantitative RT-PCR analysis of whole brain tissue at the time of euthanasia demonstrated a greater than 50% reduction in transgene expression following treatment with TMEV Xho1-OVA8 compared to controls (Fig 3G, p = 0.024). This loss of expression demonstrates effective vaccine-induced immunity targeting the OVA_257–264_-expressing glioma cells and indicates that subsequent immune editing following targeting of a single antigen contributes to the eventual outgrowth of the treated tumors.

### Functional cytotoxic activity of CD8+ T cells is required for picornavirus vaccination efficacy against GL261-Quad gliomas

**Fig 4:**
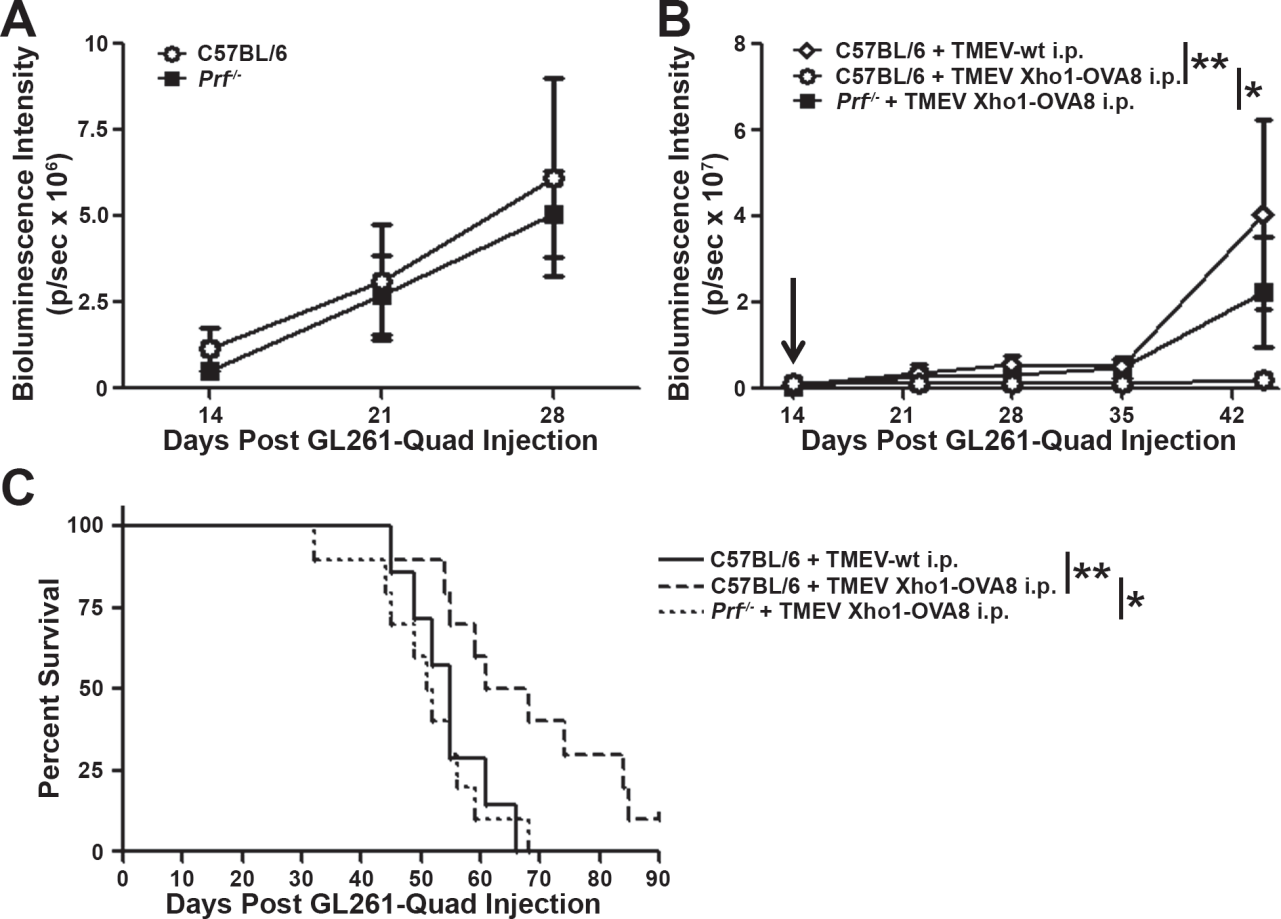
Loss of CD8+ T cell cytotoxicity abrogates the effects of picornavirus vaccination against GL261-Quad gliomas. (A) Mean bioluminescence intensity (p/sec) of wild type C57BL/6 or perforin deficient (*Prf* ^*-/-*^ *)* GL261 glioma-bearing mice demonstrates no difference in the rate of tumor progression in the absence of treatment (N = 18 mice/group). (B) Mean BLI (p/sec) and (C) survival curve of C57BL/6 (N = 10 mice/group) or *Prf* ^*-/-*^ (N = 7 mice) GL261-Quad glioma-bearing mice following picornavirus vaccination demonstrates a complete loss of vaccine efficacy in the absence of functional cytotoxic CD8+ T cell activity. Error bars indicate SEM. * denotes p<0.05, ** denotes p<0.01, ***denotes p<0.001. Arrow denotes time of vaccine administration.

The delayed tumor progression and enhanced survival of GL261-Quad glioma-bearing mice following picornavirus vaccination were accompanied by increased tumor antigen-specific CD8+ T cell infiltration into the CNS, pointing to a critical role for this immune cell type in controlling tumor burden. To directly evaluate the necessity of functional cytotoxic CD8+ T cells in delaying tumor outgrowth, experiments were repeated in mice deficient in the effector molecule perforin. Importantly, tumor progression in the absence of treatment was not found to be different in perforin deficient mice (*Prf* ^*-/-*^) compared to wild type C57BL/6 controls (Fig 4A). However, the loss of perforin completely abrogated the positive effects of picornavirus vaccination, with TMEV Xho1-OVA8 treated *Prf* ^*-/-*^ mice (N = 7 mice) displaying tumor progression and survival comparable to control mice receiving TMEV-wt (N = 10 mice) (Fig 4B and 4C). Differences in median survival of perforin deficient and TMEV-wt treated mice compared to C57BL/6 mice receiving TMEV Xho1-OVA8 (N = 10 mice) were 9.5days (p = 0.03) and 13days (p = 0.008), respectively (Fig 4C).

## Discussion

The current work is the first to demonstrate that vaccination with an engineered picornavirus expressing tumor-specific antigen is effective at delaying GL261 glioma progression and extending survival, and that these effects are dependent upon increased CNS-infiltrating cytotoxic CD8+ T cells recognizing the tumor antigen. Additionally, it incorporates parallel 3D volumetric analysis of both T2 weighted and T1 gadolinium enhanced MRI for relative comparison within rodent CNS tumors *in vivo*. The results obtained provide a foundation for future studies aimed at understanding the mechanisms by which immune responses are generated following immunotherapeutic treatment and lay the groundwork for monitoring the effectiveness of such approaches in the GL261 model system.

MRI in the clinical setting provides the highest level of confidence of any imaging modality in the diagnosis of GBM, as well as quantitative assessment of prognostic variables such as edema, gadolinium enhancement, and tumor necrosis[33–35]. Similar to human glioblastoma, analysis of both GL261 and GL261-Quad gliomas demonstrated that volumes obtained by T2 weighted MRI were significantly greater than those from T1 gadolinium-enhanced MRI[29]. Additionally, the vascular permeability within these gliomas was largely heterogeneous, as evidenced by variable leakage of gadolinium throughout the tumor area. These findings highlight the relative inaccessibility of various regions of the tumor, which remains among the most significant obstacles to effective delivery of a number of therapies[36]. Understanding the extent to which contrast enhancement is the result of inflammation entering the CNS, as well as how the lack of permeability in some regions of the tumor impedes the effectiveness of beneficial immune responses, remains to be determined. The incorporation of MRI in the assessment of rodent models of glioma provides a clinically relevant modality for evaluating these characteristics in addition to directly evaluating changes in tumor size following treatment within a single subject.

The observation of increased CNS-infiltrating CD8+ and CD4+ T cells, macrophages, and myeloid derived suppressor cells in mice bearing GL261 and GL261-Quad gliomas sets the stage for studies aimed at defining the relationships between these cell types. In both GL261 models, the CD11b^Hi^ cellular component represented the largest fraction of CD45^Hi^ cells, similar to what has been reported in human GBM[37]. The preponderance of data pertaining to the infiltration of monocytes and macrophages in human GBM, particularly MDSCs, detail the immunosuppressive role for this cell type[38–40]. Additionally, in the GL261 model, the CD4+ T cell compartment has been shown to contribute to anti-tumor immunity through promotion of Th1 and Th17 differentiation, but also serve a suppressive role through the activity of regulatory T cells[14, 41, 42]. Several studies have suggested that the effects of regulatory T cells provide the mechanism by which activated CD8+ T cells are inhibited from effectively clearing the glioma[11, 14, 43, 44]. The interactions of these immune cell subsets will be further analyzed in the GL261 model system, with the GL261-Quad model being of great value due to its expression of both MHC class I and II restricted model epitopes.

Endogenous tumor antigens have been identified in the GL261 glioma model, including EphA2 and Garc-1. However, CD8+ T cell responses toward these antigens account for only 1% of the overall tumor-infiltrating CD8+ T cell response[11, 45, 46]. It remains to be seen whether effective enhancement of this population can be achieved following vaccination. While the development of immunotherapeutic approaches targeting endogenous tumor antigens remains the primary goal in glioma models, the low frequencies of CD8+ T cells responding to these antigens presents a significant challenge to the study of T cell immunity. Understanding the mechanisms behind effective anti-tumor immune responses, including antigen processing and presentation, migration, and cytotoxic capacity, will require robust numbers of tumor-specific CD8+ T cells, for which the GL261-Quad model will again provide a powerful tool.

The findings of this study, in combination with those elucidated by Pavelko *et al*., demonstrate that picornavirus vectors are effective in promoting strong tumor-specific CD8+ T cell responses in both the periphery and the CNS[21, 22]. Most notably, the observed effects in GL261-Quad gliomas were achieved following *(1)* only a single administration of virus and *(2)* in established tumors that have been allowed to progress for two weeks. This time point is considerably later than the vast majority of immunotherapy studies, with many beginning treatment as early as 3–5 days after intracerebral GL261 injection[11, 18, 47]. The rigorous assessment of tumors by both BLI and MRI prior to treatment in the current work provides definitive confirmation of tumor establishment and adds strength to the significance of the picornavirus vaccine approach. In addition to the tumors being much larger, allowing the tumors to develop for two weeks before treating permits the tumor microenvironment, including its immunosuppressive capacity, to become well established. Despite evidence of immunosuppressive cell subsets within these tumors, the robustness of the response generated by picornavirus vaccination was sufficient to overcome the glioma microenvironment without the incorporation of additional approaches aimed at reducing immunosuppression[11]. It is of interest that, while peripheral administration of the OVA-expressing picornavirus led to the generation of fewer tumor antigen-specific CD8+ T cells infiltrating the brain than intracranial vaccination, it also produced the greatest survival benefit. This seemingly paradoxical finding may be due to defective priming of T cells elicited by intracranial vaccination. Ohlfest *et al*. recently described reduced TCR affinity and effector function of CD8+ T cells following Poly:ICLC plus OVA vaccination as the injection site approached the tumor [18]. This tumor-dependent suppression of T cell priming is likely to influence the efficacy of picornavirus-induced CD8+ T cell responses as well, providing rationale for vaccine administration distant from the tumor site. These characteristics of picornavirus vectors demonstrate an improved ability to generate effective CD8+ T cell responses in a minimally invasive manner, an important consideration for the implementation of immunotherapeutics in a clinical setting. Human picornaviruses with homology to TMEV have been identified, including Saffold Virus and Seneca Valley Virus, which could serve as viral vectors for human vaccinations[48–50]. In particular, Seneca Valley Virus has already been reported to induce no dose-limiting toxicity following systemic administration in a phase I clinical trial investigating the oncolytic effects of the virus[51].

The present study provides a model system that is uniquely applicable to the optimization of immunotherapeutics aimed at targeting established gliomas. The model developed herein provides a system in which to investigate the mechanisms governing the generation of effective immune responses, as well as fundamental interactions between the immune and nervous systems, such as presentation of CNS-derived antigens, aiding in the development of treatments for CNS disorders stemming from a wide range of etiologies.
